# Molecular Characterization of Extended-Spectrum ß-Lactamases-Producing *Escherichia coli* Isolated from a Greek Food Testing Laboratory

**DOI:** 10.3390/antibiotics14040329

**Published:** 2025-03-21

**Authors:** Aikaterini Dikaiou, Nikolaos Tzimotoudis, Daniel Sergelidis, Emmanouil Papadogiannakis, Panagiota Giakkoupi

**Affiliations:** 1 Public Health Policy Department, University of West Attica, 11521 Athens, Greece; adikaiou@uniwa.gr (A.D.); mpapadogiannakis@uniwa.gr (E.P.); 2 Hellenic Army Biological Research Center, 15236 Athens, Greece; n.p.tzimotoudis@army.gr; 3 Laboratory of Food Hygiene, School of Veterinary Medicine, Faculty of Health Sciences, Aristotel University of Thessaloniki, 54627 Thessaloniki, Greece; dsergkel@vet.auth.gr

**Keywords:** *E. coli*, extended-spectrum β-lactamases, animal source food-derived bacteria

## Abstract

**Background/Objectives:** Over the past decade, extended-spectrum beta-lactamase (ESBL)-producing *Escherichia coli* have emerged as a significant public health concern due to their potential to spread beyond clinical settings and healthy carriers, in various environments, including in animal source foods. This study seeks to investigate the molecular characteristics of ESBL-producing *E. coli* strains isolated from food of animal origin, with a focus on chromosomal typing, plasmid typing, and the description of the associated resistance genes’ genetic environment. **Methods:** Ninety-seven food of animal origin samples were tested for *E. coli* isolates resistant to cefotaxime. The resulting isolates were then subjected to antimicrobial susceptibility testing and PCR analysis to detect beta-lactamase genes. Additional assays, encompassing mating-out procedures, molecular typing utilizing Pulsed-Field Gel Electrophoresis, Multilocus Sequence Typing Analysis, and Oxford Nanopore Technology Lite whole plasmid sequencing, were also conducted. **Results:** *E. coli* was detected in 26 raw food specimens, generating a percentage of 27%. Fourteen of the current isolates (14%) were resistant to third generation cephalosporins, producing CTX-M-1, CTX-M-15, CTX-M-55, and SHV-12 beta-lactamases. The respective genes were accompanied by Insertion Sequences ISEcp1 and IS26, facilitating their transfer. Among plasmids harboring ESBL genes, representatives belonging to incI1 incompatibility group prevailed (5/8), followed by IncY and IncX3. Most plasmids proved conjugative. Diversity of molecular fingerprints of ESBL producing *E. coli* was revealed. **Conclusions:** To the best our knowledge, this study is the first to describe the molecular characteristics of *E. coli* isolates producing ESBLs sourced from foods of animal origin in Greece. The prevalence of ESBLs in our confined food collection is primarily associated with the very successful IncI1 plasmids, which were not linked to a specific *E. coli* genetic background. This lack of association confirms that horizontal plasmid transfer plays a more significant role than clonal dissemination in the spread of ESBL-mediated cephalosporin resistance.

## 1. Introduction

Over the past decade, extended-spectrum β-lactamase (ESBL)-producing Enterobacteriaceae have emerged as a significant public health concern, due to the increased global incidence of infections caused by these pathogens. Furthermore, ESBL-producing bacteria often exhibit co-resistance to multiple antibiotic classes, significantly raising the risk of treatment failure [1]. Beyond clinical settings and healthy carriers, ESBL-producing bacteria have been detected in various environments, including food, water, and both wild and domestic animals [2].

Food-producing animals are considered a primary environmental reservoir for Enterobacteriaceae that produce ESBLs [3]. This is primarily due to the acquired resistance of both symbiotic and pathogenic microorganisms to several classes of antimicrobial agents, including third-generation cephalosporins. As a result, meat and meat products have been identified as potential sources for the transmission of resistant strains or resistance genes to humans, leading to colonization or infection at the community level [4]. Numerous studies have highlighted the prevalence of Enterobacteriaceae in meat, particularly in poultry [5].

Antimicrobial-resistant bacteria originating from food-producing animals can be transmitted to humans through the consumption or handling of contaminated food containing zoonotic pathogens such as *E. coli* [6]. Transmission may also occur through the direct contact with animals or, less commonly, via environmental contamination [7]. The prevalence of Antimicrobial Resistance (AMR) in *E. coli* is shaped by multiple factors, including selective pressure from antimicrobial use in food-producing animals, the clonal expansion of resistant strains, the spread of mobile genetic elements such as plasmids, and the co-selection mechanisms that promote multidrug resistance (MDR) [8]. Monitoring the indicator commensal *E. coli* from food provides valuable insights into the reservoirs of resistant bacteria that may spread between animal populations and humans [8]. It also helps identify reservoirs of resistance genes that could be transferred to the pathogens affecting both animals and humans. This surveillance is crucial for safeguarding public and animal health [8].

Actually, plasmid typing is a valuable tool for studying the spread of specific resistance markers, such as particular antibiotic resistance genes. It helps track plasmids across diverse genetic backgrounds, supports epidemiological research, and provides insights into the potential host range of resistance genes carried by specific plasmid types [9]. In clinical epidemiology, certain plasmid types have been identified as key vehicles for transmitting ESBL and plasmid-mediated AmpC genes in isolates from food-producing animals [9]. Certain plasmid families are more commonly found in Enterobacteriaceae and play a key role in spreading specific resistance genes. For example, plasmids carrying ESBL and acquired AmpC genes are recognized as “epidemic resistance plasmids”, as they have been detected globally in Enterobacteriaceae from various sources [10].

While the growing prevalence of antibiotic-resistant foodborne pathogens is concerning, the full scope of the antibiotic resistance gene reservoir in foodborne microorganisms remains incompletely understood. Therefore, it is crucial to investigate the genetic relationships of clones and plasmids between the clinical samples and those derived from animal-sourced food products. These comparisons are vital for understanding the role of animal-sourced food in the transmission of ESBL-producing Enterobacteriaceae, which can potentially lead to human colonization and infection. This study represents the first molecular epidemiological surveillance of ESBL producers in *E. coli* from food in Greece. Sample collection was limited to a military food-testing laboratory over a one-year period and aims to identify potential reservoirs and transmission routes of MDR within the food chain.

## 2. Results

### 2.1. Resistance/Sensitivity Testing

Out of the 97 food samples, *E. coli* was detected in 26, representing 27% of the raw food samples contaminated with *E. coli*. All isolates were obtained from meat and meat products, with no *E. coli* detected in dairy products. Sixteen out of twenty-six *E. coli* isolates exhibited resistance to penicillin and susceptibility to penicillin/inhibitors combinations, cefoxitin, and ertapenem. Fourteen *E. coli* isolates, accounting for 14%, were found to be resistant to 3rd generation cephalosporins (cefotaxime, ceftazidime, ceftriaxone) and aztreonam, with eleven isolates derived from poultry and three from bovine meat (Table 1).

Ciprofloxacin and cotrimoxazole resistance prevailed amongst isolates, occurring in eleven isolates, followed by tetracycline resistance detected in eight isolates. In addition, resistance to gentamycin was detected in three isolates, resistance to amikacin in two isolates and streptomycin resistance in three isolates (Table 1).

### 2.2. Identification of β-Lactamase Producers

All fourteen isolates exhibiting the resistance to 3rd generation cephalosporins tested positive in the DDS test, confirming ESBL production. The ESBL phenotypes were associated with the presence of *bla*_CTX-M_ genes in eleven isolates. Specifically, *bla*_CTX-M-55_ (100% homology with GenBank KX889073) was detected in five isolates, *bla*_CTX-M-15_ (100% homology with GenBank AY044436) in four isolates and *bla*_CTX-M-1_ (100% homology with GenBank DQ915955) in two isolates. Furthermore, three ESBL-positive isolates possessed the *bla*_SHV-12_ β-lactamase gene (homology 100% to GenBank KF976405) (Table 1). Two penicillin resistant isolates and two CTX-M-15 producers were associated with the presence of *bla*_TEM-1B_ (100% homology with GenBank AY458016).

No AmpC or carbapenemase producers were detected.

### 2.3. Molecular Typing

PFGE revealed a diverse range of molecular fingerprints, exhibiting at least seven band differences [11]. All patterns were considered unrelated to each other, except for two CTX-M-15 producers, 389 and 390, exhibiting indistinguishable molecular fingerprints PF Type III (Table 1).

MLST of the fourteen ESBL producers identified ten types. CTX-M-1 producers were allocated into STs 1400 (10-2-3-17-18-1-4-5) and 302 (50-47-3-2-5-7-4-55). CTX-M-15 producers were classified in two different STs: ST3 (3-8-5-11-8-3-5-3) and ST716 (10-28-3-152-18-1-4-2). The five CTX-M-55 producers were classified into four different STs: two isolates identified as ST31 (20-42-21-22-19-27-19-17) and the rest as 132 (10-2-7-3-7-1-4-2), 539 (2-11-23-136-10-15-10-12) and 901 (149-3-21-194-26-213-185-40). SHV-12 producers were allocated into ST 88 (32-47-4-10-16-7-4-5), ST 416 (19-12-52-50-136-13-11-108) and ST539 (2-11-23-136-10-15-10-12) (Table 1).

Isolates 389 and 390, both CTX-M-15 producers assigned to ST 716, shared indistinguishable Pulsed Field Electrophoresis profile III. They were retrieved from chicken and bovine meat, respectively, on the same day. This confirmed that clonal similarity can be attributed to cross contamination.

Isolates 404 and 421 (CTX-M-15 producers) were both allocated into ST3, isolated from chicken and bovine meat, respectively, on different dates, and exhibited totally different molecular fingerprints PF. ST 539 was presented by isolates 422 and 402, which produced different β-lactamases, CTX-M-55 and SHV-12, respectively, were isolated both from chicken meat in different dates and exhibited different molecular fingerprints PF (Table 1).

International high-risk clones ST10 and ST131 were not detected.

### 2.4. Plasmid Characterization

**Figure 1:**
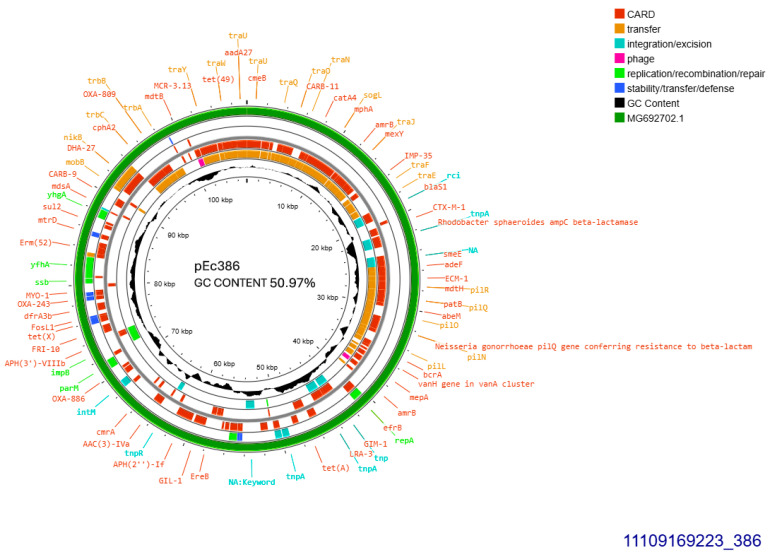
Genomic circular map of pEc386, allocated into Incl1-IA, accommodating *bla*_CTXM-1_. The predicted resistance genes are highlighted in red, the GC content is indicated in black, and the bacterial mobile genetic elements (MGEs) are represented in the colors shown in the legend. Outer track (in green) represents the sequence similarity BLAST to its closest related MG692702.1.

Both CTX-M-1 producers harbored conjugative plasmids that were transferred at a rate of 10^−6^/donor cell and were sized approximately 108 kb (Table 1). Plasmid derived from *E. coli* 386 was subjected to Whole Plasmid Sequencing. It was allocated into incompatibility group IncI1-I(alpha), pMLST3, Clonal Complex 3, exhibiting 100% identity and 100% coverage with the five respective alleles: ardA_1, pilL_2, repI1_2, sogS_1, trbA_4. The current plasmid sized 107,495 bp, with 50.97% GC content exhibited 99% homology with plasmid p644-7, GenBank MG692702.1 (Figure 1). The prototype plasmid was described in 2018, isolated from *E. coli* in free-range broilers in France [12]. In pEc386, *bla*_CTX-M-1_ was located 81 bp downstream of ISEcp1B. In addition, the plasmid harbored an intact tra operon, facilitating its conjugal transfer. Concurrently, *tet(A)* and *sul2* were both located on the plasmid (Figure 1).

In addition, three other insertion sequences were hospitalized on the current plasmid: IS26 located just upstream of ISEcp1B, and ISSbo1 and ISVsa3 located at identical orientation. The second CTX-M-1 producer, *E. coli* 384, seemed to harbor additionally a second 80 kb-sized plasmid, in S1 electrophoresis (Table 1).

**Figure 2:**
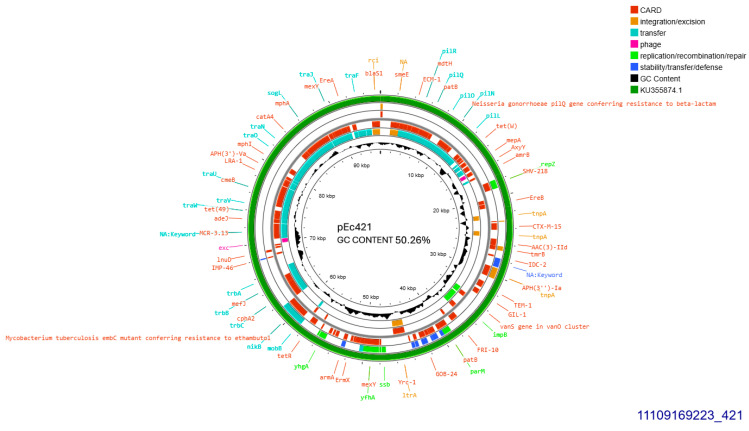
Genomic circular map of pEc421, allocated into Incl1-IA, accommodating *bla*_CTXM-15_. The predicted resistance genes are highlighted in red, the GC content is indicated in black, and the bacterial mobile genetic elements (MGEs) are represented in the colors shown in the legend. Outer track (in green) represents the sequence similarity BLAST to its closest related KU355874.1.

A single CTX-M-15 producing isolate (421), accommodated a conjugative plasmid that was transferred at a rate of 10^−5^/donor cell (Table 1). The current plasmid was subjected to Whole Plasmid Sequencing. It exhibited 99.96% similarity with plasmid pFAM22871 GenBank KU355874.1 which was isolated in Switzerland from an *E. coli* derived from dairy, in 2015 [13]. Their homology was disrupted by a 113 bp-sequence lain exclusively on pEc421 coding for a hypothetical protein. Plasmid pEc421 was sized 95,904 bp, exhibited 50.26% GC content, and was found to belong into Incompatibility Group IncI1-I(alpha), pMLST 37 (Clonal Complex 3) exhibiting 100% identity and 100% coverage with the five respective alleles: ardA_8, pilL_7, repI1_1, sogS_3, trbA_12. *bla*_CTX-M-15_ was located, 447 bp downstream of IS26. On the same plasmid, ISCfr1 and cn_3157_IS26 composite transposon were located on identical orientation. In addition, an intact *tra* operon was identified. Apart from *bla*_CTX-M-15_, *bla*_TEM-1B_ and a gene conferring resistance to gentamycin, *aac(3)-IId* were detected (Figure 2).

**Figure 3:**
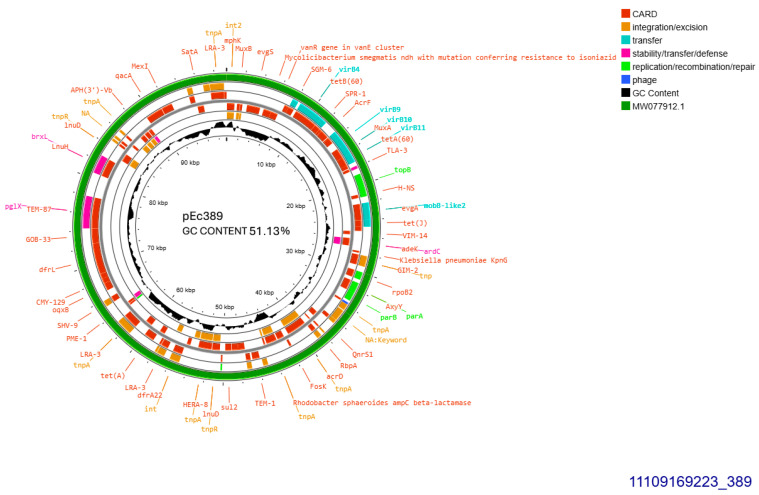
Genomic circular map of pEc389, allocated into IncY, accommodating *bla*_CTXM-15_. The predicted resistance genes are highlighted in red, the GC content is indicated in black, and the bacterial mobile genetic elements (MGEs) are represented in the colors shown in the legend. Outer track (in green) represents the sequence similarity BLAST to its closest related MW077912.1.

The two similar CTX-M-15-producing isolates 389 and 390 harbored identical plasmids sized approximately 99,305 bp, transferable by transformation. Plasmid from isolate 389 was subjected to whole plasmid sequencing, contained 51.13% GC and exhibited 93–100% similarity to plasmid pPO125 GenBank MW077912.1 (Figure 3) described in 2020 in the United Kingdom (direct submission). The current plasmid was allocated into incompatibility group IncY, and accommodated *bla*_CTX-M-15_ 49 bp downstream of ISEcp1. Additionally, four copies of IS26 were detected upstream and downstream of *bla*_CTX-M-15_. No functional *tra* operon was identified. Apart from *bla*_CTX-M-15_, seven other resistant genes were detected: *bla*_TEM-1B_, *sul2*, *aph(6)-Id*, *and aph(3″)-Ib*, both conferring resistance to streptomycin, *tet(A)*, *dfrA14* and, finally, *qnrS1* conferring resistance to ciprofloxacin (Figure 3).

Last CTX-M-15 producer, isolate 404, did not yield transconjugant or transformant and did not harbor any plasmid visible in the S1 protocol.

Four out of five CTX-M-55 producers seemed to carry non-transferable plasmids sized approximately 110 kb. A single isolate (388) displayed no visible plasmid in the S1 protocol. Resistance to many non-beta lactam antibiotics, tetracyclin, and ciprofloxacin strictly included, was detected in all isolates (Table 1). In addition, isolates 385 and 423, except from plasmid sized 110 kb, harbored also smaller plasmids.

**Figure 4:**
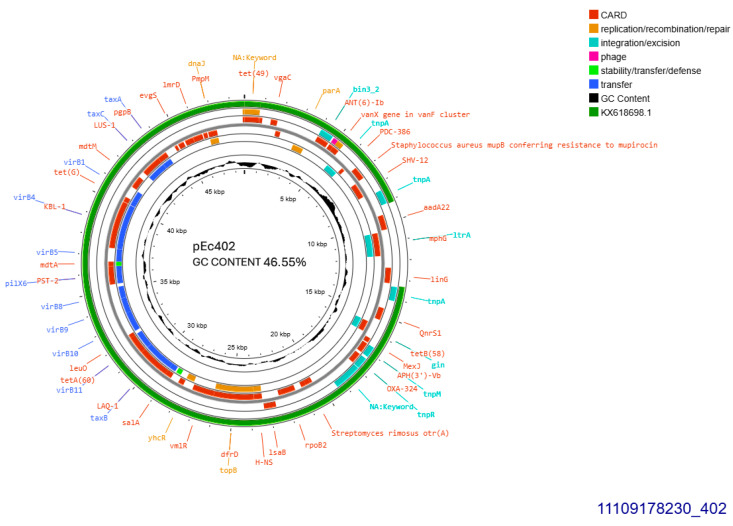
Genomic circular map of pEc402, allocated into IncX3, accommodating *bla*_SHV-12_. The predicted resistance genes are highlighted in red, the GC content is indicated in black, and the bacterial mobile genetic elements (MGEs) are represented in the colors shown in the legend. Outer track (in green) represents the sequence similarity BLAST to its closest related KX618698.1.

All SHV-12 producers, 402, 405, and 406, transferred *bla*_SHV-12_ gene through conjugation at a rate of 10^−4^/donor cell and the respective plasmids were sized approximately 50, 110, and 110 kb, respectively. pEc405 and pEc406 belonged to incompatibility group IncI1. Plasmid purified from transconjugant *E. coli* 402, was subjected to whole plasmid sequencing. pEc402 sized 48,724 bp, exhibited GC content 46.55% and was 91% similar to plasmid pEC-243 from *E. coli* 243 with GenBank KX618698.1 (Figure 4) retrieved from chicken feces in the Netherlands in 2014 [14]. Sequence blast revealed that pEc402 accommodated an approximately 4201 bp sequence that is not present on the protype plasmid pEC-243. The aforementioned sequence is coding for a class1 integron harboring *ant(3″)-Ia* family aminoglycoside modifying gene, and displaying 98% homology similarity to GenBank CP072517.1 from *Klebsiella quasipneumoniae*. Both pEc402 and pEC-243 plasmids harbored *bla*_SHV-12_ and *qnrS1*, the latter gene conferring resistance to ciprofloxacin. *bla*_SHV-12_ was part of an IS26 family composite transposon. Concurrently, pEc402 plasmid carried *aadA22* that conferred resistance to streptomycin and *inu(F)* for lincomycin resistance. Unlike both other SHV-12 bearing plasmids, pEc402 was allocated into incompatibility group IncX3 (Figure 4). An active *tra* operon was detected on the current plasmid.

## 3. Discussion

This study is, to the best our knowledge, the first to describe the molecular characteristics of *E. coli* isolates producing ESBLs sourced from animal-derived foods in Greece. Our findings align with previous studies indicating that poultry-derived foods exhibit the highest prevalence of ESBL-producing *E. coli*, while bovine and dairy products show the lowest prevalence [15].

The prevalence of ESBL-producing *E. coli* in our study was lower than that reported by the Aristotle University in Thessaloniki, Greece (AUTh), who collected neck skin specimens [16], which is much more susceptible to contamination than raw food collection. Unlike the aforementioned study, our collection included, apart from CTX-M producers, also SHV-12-producing isolates, but no AmpC producers. TEM producers were detected at a lower percentage in our study compared to northern Greece. Additionally, other non β-lactam-resistant traits were prevalent in the northern Greece study, a finding confirmed by our data, in which the *qnrS1* gene was detected for the first time in Greece. Furthermore, the prevalence of ESBL-producing *E. coli* in our collection aligns with the findings of a German study [17]. However, unlike our results, where the β-lactamases CTX-M-15 and its one-mutation-derived variant, CTX-M-55, predominated, the German study reported CTX-M-1 as the most prevalent. Additionally, other European food studies have frequently detected CTX-M-1, with its occurrence reaching up to 24% [18]. The percentage of ESBL-producing *E. coli* in food samples from our study was half that reported in a recent Spanish study [19], where *E. coli* producing CTX-M enzymes was dominant, particularly CTX-M-14. In both, our study and the Spanish study, SHV-12 was found at approximately 20%, while TEM-producing *E. coli* was detected much less frequently.

Similarly, in a study conducted by Tsekouras [20], samples were collected from suckling and weaned piglets from 34 farms in Greece and revealed that the CTX-M-1/15 enzymes were the most frequently observed ESBLs, followed by TEM and SHV types. This suggests that the prevalence of ESBL-producing *E. coli* in food is comparable to that in food-producing animals, as observed in other Greek studies [16]. The high prevalence of ESBL-producing *E. coli* in meat highlights the transmission of resistant bacteria from farm animals to food products, supporting the hypothesis that antimicrobial resistance spreads throughout the food chain.

A Multilocus Sequence Typing (MLST) analysis of ESBL-producing *E. coli* isolates revealed significant clonal diversity. Among the ten identified Sequence Types (STs), three have been predominantly linked to human infections, namely ST31 [21], ST416 [22], and ST716 [23], recognized as human pathogens. These STs may serve as potential indicators of direct transmission of resistant *E. coli* strains from food sources to humans. Other particular STs, such as ST88 [24], and ST132 [25,26] are associated with infections in both humans and food-producing animals, align with the One Health concept, highlighting their role in the interconnected spread of antimicrobial resistance across species and environments. As a matter of course, there are *E. coli* STs strictly correlated with food animals across Europe, for example, ST3 [18] and ST539 [27].

Clonal expansion of *bla*_CTX-M-15_ through ST131 has been reported in human-derived *E. coli* populations [10]. In our study, only limited clonal spread was observed via ST3 and ST716. Notably, high-risk clones such as ST131 or ST10 were not detected, reinforcing the current view that there is no clear evidence of whole-bacterium transmission of cephalosporin-resistant *E. coli* from poultry meat to humans [28].

The Incl1-IA pMLST3 plasmid was the predominant vehicle carrying the resistance genes *bla*_CTX-M-1_, *bla*_CTX_-_M-15_ and *bla*_SHV-12_ in our specimens. This highly successful plasmid is currently spreading among *E. coli* strains from both human and animal sources worldwide [10,29] underscoring its significant role in the dissemination of ESBL resistance and its potential for cross-species transmission. A recent Dutch study [30] further supports this concern, as the same plasmid was identified in *E. coli* isolates from patients with bloodstream infections and was found to be closely related to plasmids from *E. coli* of animal origin.

Localization of *bla*_CTX-M-15_ on an IncY incompatibility plasmid was recently reported in Poland in multidrug-resistant *E. coli* isolated from dairy cattle feces [31]. A similar ESBL/plasmid combination was simultaneously identified in Canada, where *E. coli* carrying the same resistance determinants was found in dairy cattle from Québec [32]. In both countries, the IncY plasmid harbored *bla*_CTX-M-15_ alongside *qnrS1*, *bla*_TEM-1_, *aph(3′)-Ib*, *aph(6)-Id*, *sul2*, *dfrA14*, and *tetA*, closely resembling our findings. This plasmid has been detected in Enterobacterales isolated from both humans and animals, emphasizing the urgent need for robust surveillance of antibiotic-resistant bacteria and the mobile genetic elements that facilitate their spread. Antibiotic resistance knows no borders, and antimicrobial resistance genes (ARGs) can transfer between bacterial strains with minimal constraints, posing a significant threat to public health.

Reports of *E. coli* producing CTX-M-55 from food or food-producing animals remain scarce in Europe. Notably, *E. coli* isolates producing CTX-M-55 from French cattle were suggested to have originated from Asia in 2018 [33]. Additionally, the Swiss Centre for Antibiotic Resistance (ANRESIS) reported the presence of CTX-M-55-producing *Shigella sonnei* in 2019 [34]. In contrast, CTX-M-55 has been widely detected in *E. coli* isolated from food animals and pets in mainland China and Hong Kong since its initial identification in ESBL-producing bacteria in Thailand in 2004 [35]. Following its first detection in Chinese clinical settings in 2010, reports of this enzyme surged, making it the second most prevalent CTX-M subtype in Chinese *E. coli* clinical isolates and the most common in *E. coli* from animals [36]. Chromosomal localization of *bla*_CTX-M-55_ cannot be ruled out, as similar events have been increasingly reported in recent years [36].

Environmental presence of *E. coli* producing CTX-M-55 was recently reported in French rivers [37], while Enterobacterales carrying this ESBL enzyme have been detected in Swiss lakes since 2013 [38]. Additionally, in rivers and wastewater treatment plants around Barcelona, Spain, Enterobacterales harboring *bla*_CTX-M-55_ and *bla*_TEM-1_, associated with IS26, have been identified [39]. These findings suggest that the prevalence of this ESBL gene in the community may be higher than currently recognized. Supporting this, *E. coli* strains carrying *bla*_CTX-M-55_ were later reported in the community of Madrid, Spain [40]. Furthermore, Portugal recently documented intestinal carriage of CTX-M-55-producing Enterobacteriaceae in patients upon hospital admission in Lisbon, further confirming the circulation of this resistance gene in the community [41].

To date, *E. coli* producing CTX-M-55 has not been reported in Greece, neither in humans nor the environment [42]. However, *Salmonella enterica Typhimurium* producing CTX-M-55 was identified in three pediatric patients in Thessaloniki, Greece, in 2022 [43]. In this case, the ESBL gene was confirmed to be chromosomally localized, similar to the findings in our *E. coli* collection, where gene localization on a non-transferable plasmid or chromosome was suggested, but not experimentally confirmed.

In our study, *bla*_CTX-M-55_ was associated with multiple *E. coli* sequence types previously linked to both human and animal infections. Its strong correlation with the tetracycline resistance aligns with the One Health concept and closely resembles Chinese strains, despite our food specimens originating from Greek farms [36]. Additionally, all *bla*_CTX-M_ derivatives are often linked to various insertion sequences (ISs), such as ISEcp1 and IS26 [36,44], which plays a crucial role in its further dissemination. This study underscores the importance of identifying transposon elements along with epidemic plasmids from a One Health perspective, emphasizing their role in the spread of antimicrobial resistance across humans, animals, and the environment.

The recent emergence of Enterobacterales bearing *bla*_SHV-12_ and *qnrS1*, encoded by IncX3 conjugative plasmids in broilers in Switzerland (2023) [45], resembles the two isolates derived from chicken meat in our collection. IncX3 plasmids were among the predominant rep-types encoding *bla*_SHV-12_ among food-producing animals and retail meat in the Netherlands from 2012 onwards [15]. The diversity of *E. coli* STs identified in the latter study reflects the complex genetic backgrounds within which *bla*_SHV-12_-harboring IncX3 plasmids circulate and provides further evidence of the successful proliferation of this plasmid subgroup. SHV-12 producing *E. coli* was recently reported in food producing animals in a slaughterhouse in central Greece [46], but the carrying plasmid was allocated into a different incompatibility group than the one stated on the current collection. In the human sector, SHV-12 is accompanied KPC-2 produced by the *Klebsiella pneumoniae*, which is responsible for epidemic status described in Greece till 2008 [47,48].

However, the plasmids pEc386, pEc389, and pEc421 displayed GC content comparable to that of the *E. coli* chromosome [49], suggesting that they are likely not foreign DNA molecules. In contrast, pEc402, which contains an insertion of a hypothetical integron segment, exhibited a lower GC content than the *E. coli* chromosome, which is consistent with an origin of the interspecies transfer.

Despite the limited number of specimens, this is the first study in Greece to characterize the molecular profile of ESBL-producing *E. coli* isolates from food. Notably, our findings do not support the direct transfer of ESBL-producing *E. coli* between humans and food of animal origin. The spread of ESBLs in Greek food from animal sources is primarily driven by plasmids, mostly belonging to a single incompatibility group. While these plasmids were not associated with a specific *E. coli* genetic background, not all were proven to be self-transferable. The lack of linkage between plasmids and *E. coli* genetic lineages further highlights that the horizontal plasmid transfer plays a more significant role than clonal dissemination in the transmission of ESBL-mediated cephalosporin resistance between food of animal origin and humans. This finding highlights the intricate relationship between human, animal, and environmental health in the spread of antimicrobial resistance, emphasizing the urgent need for comprehensive surveillance and targeted intervention strategies.

## 4. Materials and Methods

### 4.1. Bacterial Isolation and Antimicrobial Susceptibility Testing

A total of 97 samples were processed in the microbiological laboratories of the Hellenic Army Biological Research Center, over a one year period from September 2021 to August 2022. These samples consisted of raw food of animal origin, specifically poultry meat 34 samples, bovine meat 27 samples, porcine meat 11 samples, as well as 25 dairy products, mainly cheese. The meat and poultry products were processed in approved facilities (cutting plants of minced meat and meat preparation as well as poultry) and were collected upon delivery to the catering establishment. Similarly, dairy products were processed and packed in approved facilities and distributed to catering establishments through authorized cold storage facilities. Sampling was conducted following the established guidelines for microbiological analysis as set by European Commission Regulation (EC) No 2073/2005. Each sample was collected, transferred, and stored at temperatures between 0 and 5 °C prior to processing.

According to the aforementioned EC, approximately 25 g of each sample were combined with 225 milliliters of Buffered Peptone Water (Biolab, Budapest, Hungary) and incubated at 37 °C for 18 to 20 h, as part of the analysis for detection of *Salmonella* spp. according to ISO 6579-1:2017 [50]. Following enrichment, the cultures were plated onto TBX agar (Oxoid, UK) and incubated at 44 ± 0.5 °C for 18 to 20 h, according to ISO 16649-2:2001 [51]. Colonies exhibiting a blue color due to β-D-glucuronidase activity were identified as *E. coli*. Identification was further confirmed using the indole test (at 44 ± 0.5 °C) and the methyl red, Voges–Proskauer, and citrate tests (at 37 °C). From each sample, one plate was obtained, and an average of six colonies were retrieved per plate, which were then subjected to antibiotic resistance/susceptibility testing. Isolates resistant to 3rd generation cephalosporins were subjected to further analysis. Antibiotic disks used were purchased from OXOID Ltd. (Basingstoke, UK), G & M Procter Ltd. (Perth, UK), Thermo Fisher (Heysham) Limited (Heysham, UK), Oxoid Limited (Ireland) (Dublin, Ireland) and were as follows: ticarcillin 75 μg, piperacillin 30 μg, piperacillin-tazobactam 36 μg, amoxicillin-clavulanic acid 30 μg, cefoxitin 30 μg, cefotetan 30 μg, cefotaxim 5 μg, ceftazidime 10 μg, cefepim 30 μg, aminoglycosides (gentamicin 10 μg, amikacin 30 μg), cotrimoxazole 25 μg, tetracycline 30 μg, chloramphenicol 30 μg, and ciprofloxacin 5 μg, following EUCAST guidelines [52].

In cases of phenotypic resistance to ceftazidime and/or cefotaxime, a double disk synergy test was performed to detect ESBL production. Isolates exhibiting cefoxitin resistance were further tested with and without 250 mg/L cloxacillin to detect AmpC β-lactamase production. Both phenotypic tests are recommended by EUCAST guidelines for detecting resistance mechanisms of clinical and epidemiological importance (Version 2.01, July 2017).

### 4.2. Genotypic Characterization of Resistance Determinants

DNA extraction was carried out using the Nucleospin Tissue kit from Macherey Nagel GmbH & Co. KG (Düren, Germany). ESBL-encoding genes were identified through PCR with previously published primers [53], followed by sequencing. The resulting PCR amplicons underwent a Sanger sequencing analysis (CeMIA SA, Larisa, Greece, https://cemia.eu/service/sanger-sequencing/, accessed on 14 October 2023), as described previously [42].

### 4.3. Mating-Out Assays and Plasmid Analysis

A liquid-mating assay with *E. coli* 1R716 streptomycin-R lac (−) and *E. coli* 39R793 rifampicin-R lac (-) as recipients investigated the transferability of resistant determinants, as described previously [53]. Transconjugants were selected from the McConkey agar supplemented either with streptomycin (2000 mg/L), or rifampicin 100 mg/L along with cefotaxime or ceftazidime (1 mg/L).

Nucleobond Xtra MIDI, Macherey Nagel GmbH & Co. KG (Düren, Germany) was employed for plasmid purification to transform DH5a competent cells, prepared manually according to Molecular Cloning Laboratory Manual, Second Edition, J. Sambrook, E.F. Fritsch, T. Maniatis. [54]. Transformants were selected on LB agar supplemented with 1 mg/L ceftazidime or cefotaxime. Transconjugants and transformants were subjected to PCR for confirmation and antimicrobial susceptibility testing. The presence of a single plasmid in each transconjugant or transformant was verified through digestion with S1 nuclease (ThermoFisher Scientific Inc., Waltham, MA, USA), an endonuclease that introduces single-stranded nicks and breaks in duplex DNA, converting circular plasmids into linear forms [55]. Plasmid size was determined using pulsed-field gel electrophoresis (PFGE) [55].

Plasmid DNA from transconjugants and transformants was purified using the Nucleospin Plasmid MIDI kit (Macherey Nagel GmbH & Co. KG, Düren, Germany). Additionally, all transconjugants and transformants underwent PCR-based replicon typing (PBRT), following previously described protocols [56].

Representative plasmid codings for each beta-lactamase were subjected to Oxford Nanopore Technology (ONT) Lite Whole Plasmid Sequencing, conducted by Eurofins. ONT was used to obtain very long sequences of several kilobases. Sequencing and the resulting reads were then subjected to quality filtering, assembly, and annotation using the Nanopore data analysis pipeline (31 December 2020). The draft sequence of these plasmids was used for the characterization of the β-lactamase genetic environment.

Sequence similarity search was performed using BLAST v1.4.1 (https://blast.ncbi.nlm.nih.gov/Blast.cgi, accessed on 15 July 2024) scans against the GenBank database. Acquired ARGs and other features in the plasmid DNA of each isolate were identified using the websever ResFinder 4.1 (http://genepi.food.dtu.dk/resfinder, accessed on 10 December 2024) with a minimum coverage of 80% and a minimum identity of 95% as well as Proksee software. The PlasmidFinder bioinformatic tool (https://cge.food.dtu.dk/services/PlasmidFinder/, accessed on 18 March 2025) was used for the identification of plasmid replicon types (incompatibility groups).

The PROKSEE server (https://beta.proksee.ca, accessed on 18 March 2025) was used to generate high-quality navigable maps of each circular plasmid [57]. Plasmids’ genome sequence was retrieved from NCBI in GenBank format using their accession numbers and submitted to Proksee. A variety of tools/databases was used for the generation of the graphical map were then used within Proksee: Comprehensive Antibiotic Resistance Database (CARD, v1.2.1) Resistance Gene Identifier (RGI) is used to predict the antibiotic resistome(s) from protein or nucleotide databased on homology [58], the mobileOG-db (v1.1.3) which tracks the mobile genetic elements (MGEs) [59], BLAST which compares the given sequence with another genome sequence [60], and the GC tool which adds a GC Content plot (v1.0.2). When the output from these analyses is added to the map a region supported by all tools is apparent.

### 4.4. Clonality Evaluation

Pulsed-field gel electrophoresis (PFGE) was used to evaluate the clonal relationship of E. coli isolates. Total DNA was digested with the XbaI enzyme (New England BioLabs, Ipswich, MA, USA), and the resulting fragments were separated using a CHEF-DR III System (Bio-Rad, Cressier, Switzerland) [42]. Molecular fingerprint comparisons were conducted visually following the criteria established by Tenover in 1995 [11]. Additionally, multilocus sequence typing (MLST) was performed on ESBL-producing isolates according to the Pasteur scheme (https://bigsdb.pasteur.fr/cgi-bin/bigsdb/bigsdb.pl?db=pubmlst_ecoli_isolates, accessed on 18 March 2024).

## Figures and Tables

**Table 1:** Characteristics of the *E. coli* isolates obtained from food specimens.

I/N	Sample No	Source	Isolation Date	β-Lactamase	ST	PF-Type	Conjugation Rate	Transformation	S1-Size (kb)	PBRT (ESBL)	Other Resistant Traits (Bold Are Transferred)	Genes on Plasmid
1	384	Bovine meat	11 October 2021	CTXM-1	ST1400	Ι	10^−6^	ND	80 **108**	Incl1	**SXT**, CIP	
2	386	Chicken meat	15 February 2022	CTXM-1	ST302	ΙΙ	10^−6^	ND	80 **107,495**	Incl1-IA	**TET**	***tet(A)***, ***sul2***
3	389	Bovine meat	30 November 2021	CTXM-15 TEM-1B	ST716	III	NO	YES	**99,305**	IncY	**SXT**, **TET**, **CIP**, **STR**	***sul2***, ***aph(6)-Id***, ***aph(3″)-Ib*** ***qnrS1***, ***tet(A)***, ***dfrA14***
4	390	Chicken meat	30 November 2021	CTXM-15	ST716	III	NO	YES	**99,305**	IncY	**SXT**, **TET**, **CIP**, **STR**	
5	404	Chicken meat	11 July 2022	CTXM-15	ST3	IV	NO	NO	95		GN	
6	421	Bovine meat	10 August 2021	CTXM-15 TEM-1B	ST3	V	10^−5^	ND	**95,904**	Incl1-IA	SXT, **GN**, CIP	***aac(3)-IId***
7	385	Chicken meat	15 February 2022	CTXM-55	ST132	VI	NO	NO	110 80		SXT, TET, AK, C, CIP	
8	387	Chicken meat	22 February 2022	CTXM-55	ST901	VII	NO	NO	110		SXT, TET, GN, C, CIP	
9	388	Chicken meat	29 November 2021	CTXM-55	ST31	VIII	NO	NO	ND		TET, CIP	
10	422	Chicken meat	30 August 2022	CTXM-55	ST539	IX	NO	NO	110		SXT, TET, C, CIP	
11	423	Chicken meat	29 August 2022	CTXM-55	ST31	X	NO	NO	50 60 80 110		SXT, TET, AK, C, CIP	
12	402	Chicken meat	15 June 2022	SHV-12	ST539	XI	10^−4^	ND	**48,724**	IncΧ3	**STR**, **CIP**	***aadA22***, ***inuF***, ***qnrS1***
13	405	Chicken meat	24 June 2022	SHV-12	ST88	XII	10^−4^	ND	100 **110**	IncI1	**SXT**, **TET**	
14	406	Chicken meat	26 July 2022	SHV-12	ST416	XIII	10^−4^	ND	**110**	IncI1	-	
15	211842IV	Bovine meat	10 August 2021	NO	ND	ND	ND	ND	ND	ND	-	ND
16	212074III	Bovine meat	14 September 2021	NO	ND	ND	ND	ND	ND	ND	-	ND
17	212112I	Bovine meat	16 September 2021	NO	ND	ND	ND	ND	ND	ND	-	ND
18	212113III	Bovine meat	16 September 2021	NO	ND	ND	ND	ND	ND	ND	-	ND
19	212144	Bovine meat	21 September 2021	TEM-1	ND	ND	ND	ND	ND	ND	TIC, SXT	ND
20	2122271I	Chicken meat	4 October 2021	TEM-1	ND	ND	ND	ND	ND	ND	TIC, SXT, CIP	ND
21	2122272I	Bovine meat	4 October 2021	NO	ND	ND	ND	ND	ND	ND	-	ND
22	211476	Bovine meat	23 August 2021	NO	ND	ND	ND	ND	ND	ND	-	ND
23	212072	Chicken meat	14 September 2021	NO	ND	ND	ND	ND	ND	ND	-	ND
24	212763	Porcine meat	30 November 2021	NO	ND	ND	ND	ND	ND	ND	-	ND
25	220239	Chicken meat	15 February 2022	NO	ND	ND	ND	ND	ND	ND	-	ND
26	220240	Bovine meat	15 February 2022	NO	ND	ND	ND	ND	ND	ND	-	ND

Abbreviations: SXT = Trimethoprim/sulfamethoxazole, CIP = Ciprofloxacin, TET = Tetracycline, STR = Streptomycin, GN = Gentamycin, AK = Amikacin, C = Chloramphenicol, TIC = Ticarcillin, ND: not determined.

## Data Availability

GenBank access numbers for nucleotide sequences: PV253946 for pEc386, PV253947 for pEc421, PV253948 for pEc389 and PV253949 for pEc402.
